# A novel small-molecule SIRT1 inhibitor induces apoptosis in leukemic cell lines

**DOI:** 10.1186/1742-4690-11-S1-P86

**Published:** 2014-01-07

**Authors:** Tomohiro Kozako, Takayoshi Suzuki, Makoto Yoshimitsu, Shohei Itonaga, Akiyoshi Aikawa, Shin-ichiro Honda, Naomichi Arima, Shinji Soeda

**Affiliations:** 1 Department of Biochemistry, Faculty of Pharmaceutical Sciences, Fukuoka University, Fukuoka, Japan; 2 Graduate School of Pharmaceutical Sciences, Nagoya City University, Nagoya, Aichi, Japan; 3 Division of Hematology and Immunology, Center for Chronic Viral Diseases, Graduate School of Medical and Dental Sciences, Kagoshima University, Kagoshima, Japan

## 

ATL is an aggressive peripheral T-cell neoplasm that develops after long-term infection with HTLV-1. The development of new treatment with ATL is desired. SIRT1, a nicotinamide adenine dinucleotide (NAD+)-dependent histone/protein deacetylase, plays a crucial role in various physiological processes, such as aging and apoptosis, owing to its ability to deacetylate numerous substrates. Here, we assessed how SIRT1 is regulated in primary ATL cells and leukemic cell lines. SIRT1 expression in ATL patients was significantly higher than that in healthy controls. NCO-01 and NCO-04, novel small-molecule SIRT1 inhibitors, induced significant growth inhibition or apoptosis in leukemic cell lines, especially S1T cells, which is HTLV-1-infected CD4+ T cells derived from an ATL patient with no Tax expression. NCO-01 and NCO-04-induced apoptosis was mediated by activation of the caspase family. Lack of SIRT1 expression also increased the apoptosis of MT-2 cells. These results suggest that SIRT1 is a crucial anti-apoptotic molecule in ATL cells. Therefore, methods to block the function of SIRT1 may be useful in developing ideal therapeutic agents for leukemia, especially in patients with ATL.

